# CALIMA: The semi-automated open-source calcium imaging analyzer

**DOI:** 10.1016/j.cmpb.2019.104991

**Published:** 2019-10

**Authors:** F.D.W. Radstake, E.A.L. Raaijmakers, R. Luttge, S. Zinger, J.P. Frimat

**Affiliations:** a Department of Electrical Engineering, Signal Processing Systems Group, Eindhoven University of Technology, Eindhoven, the Netherlands; b Department of Electrical Engineering, Electromagnetics Group, Eindhoven University of Technology, Eindhoven, the Netherlands; c Department of Mechanical Engineering, Neuro-Nanoscale Engineering Group, Microsystems Section & ICMS Institute for Complex Molecular Systems, Eindhoven University of Technology, the Netherlands

**Keywords:** Calcium imaging, Segmentation, Ca-spike detection, Neuronal network reconstruction

## Abstract

**Background and objective:**

Ever since its discovery, calcium imaging has proven its worth in discovering new insights into the mechanisms of cellular communication. Yet, the analysis of the data generated by calcium imaging experiments demands a large amount of time from researchers. Tools enabling automated and semi-automated analysis are available, but often they allow automating only a part of the data analysis process. Therefore, we developed CALIMA (https://aethelraed.nl/calima), a free and open-source standalone software tool that provides an opportunity to quickly detect cells, to obtain the calcium spikes, and to determine the underlying network structure of neuronal cell cultures.

**Methods:**

Owing to the difference of Gaussians algorithm applied for the cell detection, CALIMA is able to detect regions of interest (ROIs) quickly. The z-scoring algorithm provides a means to set the requirements for spike detection, and the neuronal connections can be reconstructed by analyzing the cross-correlation between the cellular activity. We evaluated CALIMA's reliability, speed, and functionality with a special focus on neuronal cell detection and network reconstruction. The evaluation was performed by using real-life data such as a known example dataset (cultured primary rat cortical neurons, University of Pennsylvania) and by analyzing video graphic footage of in vitro brain cell samples (SH-SY5Y neuroblastoma cultures, one sample with synchronous neuron firing). The obtained results were compared to the corresponding outcomes observed on same datasets for other similar software solutions. Moreover, we compared the results of segmentation and peak detection analysis, the ones obtained using CALIMA and those acquired manually.

**Results:**

CALIMA was able to detect the cells in the cultures within seconds. The average sensitivity was 82% across the datasets checked, comparing favorably with the alternative software solutions. Using the correct parameters, CALIMA's Ca-spikes detection sensitivity reached 96%. Lastly, neuronal networks were reconstructed by combining the data on the ROI's activity and the cell's positions, finding the most likely inter-cell connections.

**Conclusions:**

We found that CALIMA proved to be a robust and fast tool to analyze the data of experiments for the digital reconstruction of the neuronal cellular network while being able to process the analysis steps with minimal user input required and in a time efficient manner.

## Highlights

• Although calcium imaging is widely used in biological research, there are relatively few tools available for its analysis. These tools often enable only part of the analysis or require expensive software packages to run. Here we proposed the semi-automated standalone open-source software tool CALIMA, and we show that it is a valuable addition to the field of calcium imaging. As demonstrated with three highly different real-life datasets, CALIMA can detect cells and their activity with high sensitivity. CALIMA furthermore enables the user to reconstruct a network, and allows the user to limit the length of neuronal connections in said network. As some cell types grow neurites of limited length, the latter option can help to study the communication in groups of such cells.

## 1 Introduction

Communication between adjacent neurons can be investigated through calcium (Ca) imaging. When this technique is applied, the cells are treated with a Ca-sensitive fluorescent dye. Consequently, events related to the release of calcium such as inter-neuronal communication can be tracked by fluorescence microscopy. This technique has proven its worth because with its help researchers have managed to explain the communication mechanism between muscle cells, heart cells, and neurons [1].

Besides providing insights into various biological processes, this method can help in the understanding of diseases such as cancer, diabetes, or autoimmune diseases [2].

The behavior of neuronal circuits depends not only on the properties of its cells, but also on the network topology [3]. Hence, the knowledge of the connections within a neuronal network and its spatiotemporal properties can provide an insight into gene activation patterns, physiological responses, and pivotal cellular decisions [4].

While the technique can be applied on a wide range of cells for a variety of purposes, the required quantitative analysis is challenging. Namely, the cell bodies, so-called regions of interest (ROI), need to be determined, the calcium spikes detected, and a network of interacting cells constructed. While this can be done manually, it is a resource- and time-consuming task, especially given the large amount of data that is generally involved in computations. Hence, automation of the analysis is deemed helpful.

Although there are software solutions that allow automating the data analysis from an image stack, these solutions are often based on expensive software packages such as MATLAB [5], [6], [7], [8] or automate only the cell segmentation and/or the identification of calcium spikes, and they lack the functionality to detect any networks of interacting cells [9], [10], [11], although the identification of such networks is of paramount importance when working with neurons [12]. Some data types need to be pre-processed before they can be analyzed [7], [13], and some tools are handled through scripting [10], which can cause difficulties for a novice programmer. Furthermore, to finalize the analysis, it often requires a decent amount of time and a detailed knowledge of a system in question [5].

**Fig. 1:**
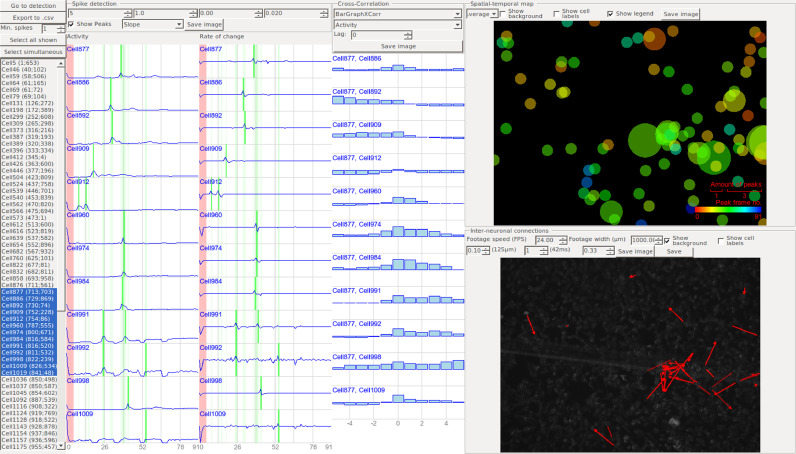
Screenshot of CALIMA GUI showing the activity and rate of change for selected ROIs and cross-correlation between selected ROIs (left), a bar graph of the cross-correlation between selected cells (middle), a spatiotemporal map of the neuronal spikes (top right, circle size is proportional to the amount of activity of a ROI, while color indicates the average). The reconstructed neuronal network is overlaid on the footage (bottom right). Red arrows represent detected neuronal connections and their direction of communication.

To aid in the analysis of the data required for calcium imaging, we developed a free and open-source standalone software tool: CALcium IMaging Analyzer (CALIMA, https://aethelraed.nl/calima), with the interface GUI shown in Fig. 1. To evaluate the reliability, speed, and functionality of CALIMA, we used the experimental data obtained from primary rat cortical neurons [14] and in-house SH-SY5Y neuroblastoma cultures [15]. The ROIs and Ca-spike detection outcomes were validated against the results generated by a trained biologist, and several falsely detected or undetected cells and spikes were observed. Moreover, the output of the proposed data analysis method was compared to the output obtained by using alternative segmentation strategies available within various software solutions, viz. Active Contours (FluoroSNNAP) [13], Otsu thresholding and intensity-based declumping (CellProfiler) [11], Thresholding and Watershedding (ImageJ) [16], and spatiotemporal independent component analysis (SIMA) [10]. The Ca-spikes detection results were compared to the results of an algorithm, which implies comparing spike shapes with a database values to determine the ROI activity (FluoroSNNAP) [13].

## 2 Theory

**Fig. 2:**
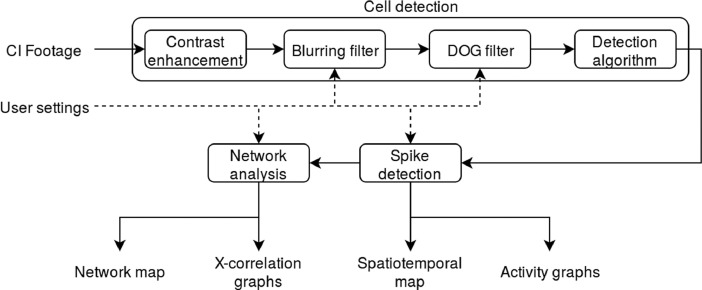
Flowchart of the CALIMA algorithm.

Several distinct stages are defined to process an image set using CALIMA as shown in Fig. 2. As a starting step, the ROIs are detected based on the area covered by cells. Next, the calcium-based activity spikes are identified per each ROI. Lastly, the generated data are analyzed to reconstruct physically possible inter-cell connections. The sections below describe these steps in more detail. The process starts by loading a file or files of calcium imaging experiments. Images can be loaded in such formats as .png, .jpg, .bmp, .gif, or .tiff (no tiff-stacks). Loading video files is enabled using FFmpeg [17], and the tool supports many formats including .avi, .wmv, and .mp4. The ROIs and analysis results can be exported as images or, in the case when the measured cell brightness and the detected communication network are also available, as .csv files.

### 2.1 Cell detection

First, the user selects a representative frame of a video or an image set, which is used as a reference to detect the cells. To make the brighter cells distinguished against the dark background, the contrast is stretched first according to Eq. (1):

(1) $$I_{x,y}=\frac{O_{x,y}-\text{min}\left(O\right)}{\max\left(O\right)-\text{min}\left(O\right)}.$$

Here the brightness of a certain pixel of the resulting image **I** at location (*x,y*) is determined as a function of the brightness of the same pixel on the original image **O** and the maximum and minimum brightness values that occur in the image. To filter out the weak inter-neuronal connections and noise, a Gaussian blurring filter is convolved with the frame image as described in Eq. (2) to obtain the blurred version **B** of the image as described in Eq. (3):

(2) $$G_{x,y}\left(\sigma \right)=\frac{1}{2\pi \sigma^{2}}\text{exp}\left(-\frac{x^{2}+y^{2}}{2\sigma^{2}}\right)$$

(3) $$B=I*G\left(\sigma_{n}\right)$$

The user-defined standard deviation σ_n_ is used as the 2D standard deviation of the blurring filter. Subsequently, a difference of Gaussians (DOG) filter, the subtraction of the same image modified by two different Gaussian filters, is applied to the resulting image **I** as described in Eq. (4) to separate the fluorescing cells from the darker background [18]:

(4) $$D\left(\sigma_{a},\sigma_{b}\right)=B*G\left(\sigma_{a}\right)-B*G\left(\sigma_{b}\right)$$

where the resulting image **D** is defined as a function of the user-defined standard deviations σ_a_ and σ_b_. In the resulting image, ROIs tend to be of a lighter shade than the background. Therefore, the image is compared to a user-defined brightness threshold *th* to determine, which pixels belong to the ROI in question. On the resulting binary image, a recursive function is used to identify clusters of adjacent pixels belonging to ROIs. To account for possible variations in the input data, of which the most important are zoom level, resolution, brightness and focus, all four parameters of the above-mentioned functions can be adjusted by the user, namely, the standard deviations of the three Gaussian filters and the threshold. For cases where the automatic detection does not suffice, it is also possible to add or remove ROIs manually. To speed up the ROI detection process, the aforementioned filters are separated and a separable convolution algorithm is implemented [19]. The processing speed of the convolution is further improved by using a multithreaded implementation where computation is distributed across several processor cores.

### 2.2 Spike detection

The brightness value of a ROI is related to the cell activity, but the base brightness varies greatly per each cell; for example, if two cells are both inactive, one may appear much brighter than another. To account for this effect, the rate of change in brightness per ROI is used for calcium spike detection instead of the brightness itself, as this is not affected by different mean brightness values [9]. This indicator is then used to detect calcium spikes via the z-scoring algorithm [20].

For each ROI and for each frame *n*, the rate of change in brightness **r**[n] = **b**[n] - **b**[n-1] is calculated. The mean brightness *µ_l_* and standard deviation *σ_l_* are calculated within a temporal window of a user-defined length *l*, according to Eqs. (5) and (6). Another user-defined parameter *i* limits the influence of spikes on the calculation of µ_l_ and *σ_l_* according to Eqs. (7) and (8) to increase the robustness of the algorithm [21].

The z-score is then defined according to Eq. (9). Such frames, for which ***z*** is greater than the user-defined threshold *th_z_*, and **r** is greater than the user-defined threshold *th_r_*, are detected as calcium spikes in the resulting vector ***s***, as described in Eq. (10). The user can compensate for spontaneous activity by setting the required minimum number of spikes detected for a ROI to be included in the analysis.

(5) $$\mu_{l}\left[n\right]=\frac{1}{l}\sum_{k=n-l-1}^{n-1}r\left[k\right]$$

(6) $$\sigma_{l}\left[n\right]=\sqrt{\frac{1}{l}\sum_{k=n-l-1}^{n-1}{\left(r\left[n\right]-\mu_{l}\left[n\right]\right)}^{2}}$$

(7) $$r_{f}\left[n\right]=\left\{ \begin{array}{c} r_{i}\left[n\right],\;if\;s\left[n\right]=1\;or\;if\;\frac{-r\left[n\right]-2\mu_{l}\left[n-1\right]}{\sigma_{l}\left[n-1\right]}>z_{th}\\ r\left[n\right],\;otherwise\; \end{array}\right.$$

(8) $$\text{Where}\;r_{i}\left[n\right]=i\cdot r\left[n\right]+\left(1-i\right)\cdot r_{f}\left[n-1\right],$$

(9) $$z\left[n\right]=\frac{r\left[n\right]-\mu_{l}\left[n-1\right]}{\sigma_{l}\left[n-1\right]}$$

(10) $$s\left[n\right]=\left\{ \begin{array}{c} 1,\;if\;r\left[n\right]>th_{r},z\left[n\right]>th_{z}\\ 0,\;otherwise\; \end{array}\right.\;$$

### 2.3 Correlation maps

After identification of the ROI activity and peaks, the Pearson cross-correlation x_τ_ for a lag τ between the ROIs is determined according to Eq. (11), where µ_f_ and µ_g_ denote the mean values of ***f*** and ***g*** according to Eq. (12). The variables **f** and **g** can be either the brightness **b**, the brightness rate of change **r**, or the spike vector **s** of ROIs as described in Section 2.2.

(11) $$x_{\tau }\left[n\right]=\sum \frac{f\left[n-\tau \right]-\mu_{f}}{\left|\left|f-\mu_{f}\right|\right|}\cdot \frac{g\left[n\right]-\mu_{g}}{\left|\left|g-\mu_{g}\right|\right|};$$

(12) $$\mu_{y}=\frac{1}{\left|y\right|}\;\sum y\left[n\right],\;y\in f,g$$

The detected peaks, the rate of change, and the activity are shown directly in a graph for separate ROIs (Fig. 1, left), whereas *x_τ_* between all combinations of selected cells can be presented in a form of bar graph for −5≤ τ ≤ 5, a correlation matrix or a correlation map (Fig. 1, center). The correlation matrix displays *x_τ_* so that the cross-correlation between the first and second selected cells is at the position (1, 2), between the first and third at (1, 3), etc., with the color of each pixel selected from a provided colormap based on the value of *x_τ_* (leftmost color for *x_τ_* = −1 up to the rightmost color for *x_τ_* = 1). This provides the user with information regarding the overall correlation between all selected ROIs.

The correlation map shows a spatial map of all detected ROIs colored depending on *x_τ_*. The single-cell correlation map displays through colors which areas show activity similar to a selected cell, providing a clear view of which ROIs have the calcium fluorescence behavior similar to the one of the selected cell. Furthermore, a heat map can be created to see which ROIs and/or areas of the cell culture are the most active. This can be seen in a spatial map of all detected ROIs colored depending on the value of **b, r** or **s** (the leftmost colormap value for 0, the rightmost color for the maximum value of **b, r** or **s**).

Another map is drawn to provide information regarding the spatiotemporal characteristics of the ROIs (Fig. 1, top right). It displays a spatial map of all ROIs, for which calcium spikes were detected, drawn as circles. The diameter of these circles is linearly dependent on the number of detected spikes for that ROI, whereas the color depends on the average spike frame *n_avg_* = ∑*s*[*n*] · *n*, ranging from red for the first frame to blue for the last one.

### 2.4 Network analysis

As a final step, the inter-neuronal connections are reconstructed. The cross-correlation between ROIs is used as a measure of the likeliness of the connection presence. A minimum correlation factor, ranging from 0.0 to 1.0, is given by the user so that only those areas that produce similar spiking patterns are considered as being functionally connected. Furthermore, a maximum connection length of the neurites can be given as input for anatomical connections (along with the image width). In that case, only connections shorter than the upper limit are considered. This feature allows the user to either include or exclude anatomical (physical) connections of the network in the analysis and provides a means to separate between functional and anatomical connections. Functional network is obtained by setting connection length to max value so that the network is based using only the correlation of activity time series. A stricter anatomical network can also be obtained by restricting the connection length value between two ROI preventing the program from reconstructing networks with connections of such length that they are not physically possible. The same accounts for the maximum delay between an incoming spike and the cellular response, which can be tuned after setting the footage frames per second (FPS). The connections are presented on a map of the cell culture (Fig. 1, bottom right), showing which ROIs are likely to be connected in physiological response. Lastly, the produced graphs and representations of the connections between the cells can be exported as a csv file for further external analysis.

## 3 Methods

The performance of CALIMA was measured on three different datasets, two of which were produced in-house, as described in Section 3.1. Both cell detection (Section 3.2) and combined spike detection were tested and benchmarked against several alternative cell segmentation and spike detection strategies as described in Section 3.3.

### 3.1 Cell culture and PDMS preparation

The neuroblastoma cell line SH-SY5Y [22] was used to characterize the performance of CALIMA software on polydimethylsiloxane (PDMS) substrates. PDMS and a cross linking agent were mixed in a cup in a ratio of 10:1 and degassed for approximately 20 min. After degassing, the liquid PDMS was poured onto a glass slide directly from the cup. Thereafter, the PDMS was cured on a hotplate at 95 °C for 10 min and coated with 50 µg/ml fibronectin solution in phosphate-buffered saline (PBS) for 45 min prior to cell seeding. SH-SY5Y neuroblastomas were cultured in DMEM/F-12 media (1:1) supplemented with 10% fetal bovine serum (FBS) and 1% pen/strep, and they were grown in an incubator at 37 °C, 5% CO_2_. When cell confluency was reached, trypsin (x1) was used to harvest the cells and thereafter, was centrifuged at 900 rpm for 5 min. A concentration of 200,000 cells/ml was used throughout the seeding experiments. Once the cells were seeded, they were left to adhere for 24 h following exposure to retinoic acid, thereafter, for 3 days at 10 µM in DMEM/F-12 media to differentiate the cells into neurons according to the manufacturer protocol [23]. Following 3 days of differentiation, a standard culture medium using DMEM/F-12 medium was added. For calcium staining experiments, the Fluo-4 Calcium Imaging kit (molecular probes, life technologies) was used according to the manufacturer's protocol and as described by [24]. Video footage of the samples were recorded using EVOS FL microscope (ThermoFisher, Waltam, MA, USA) at 1 frame per 10 s, and for 10 min with frames of 1280 by 960 pixels. When neurons maturate they become electrically active and start to blink in synchrony (coherent neuronal activity patterns) [25], a phenomenon that can be recorded by calcium imaging experiments [26]. One dataset (Dataset 1) was taken from an early differentiated neuron culture (10 days in culture), and it did not exhibit this phenomenon. Another dataset (Dataset 2) was taken from a late differentiated neuron culture (28 days in culture); for this one the effect of synchronous neuron firing was observed.

In addition to the data obtained as described, we analyzed a validated example, Dataset 3, which was provided by the FluoroSNNAP software (spontaneous activity of cultured primary rat cortical neurons transduced with AAV2-GCaMP6, 696 by 520 pixels, 10 Hz measurements for 2 min, downloaded from [14]).

**Fig. A.1:**
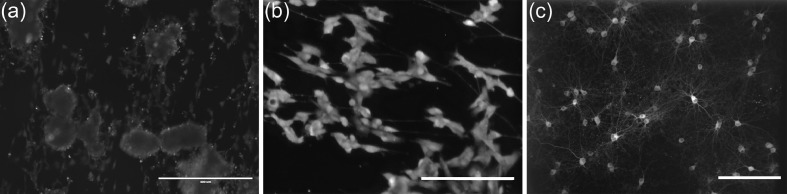
The averaged frame of each dataset. Dataset 1: A slightly active culture containing a mix of various cell types, different cell sizes and aggregates. The bar represents 400 µm (a). Dataset 2: An active culture consisting of densely-packed cells. The bar represents 200 µm (b). Dataset 3: An active culture consisting of separated cells. The bar represents 250 µm(estimated) (c).

As a result of the experimental procedures, it was observed that Dataset 1 consisted of zoomed-out densely packed barely active cells of various types and some cellular debris and Dataset 2 of zoomed-in densely packed active cells, likely connected in a network. Dataset 3 showed zoomed-in cells communicating in a network [13] located at a distance from each other. Examples of single frames from each dataset are shown in Fig. A.1.

### 3.2 Cell detection

The aforementioned neuronal cultures were with CALIMA for estimation purposes. The parameters used for cell detection varied between the datasets, the detailed description is provided in Table B.1 for reference.

The cells as detected by the softwares were validated against a manual segmentation done by a trained biologist. It was checked how many of the total number of areas that were found manually (P) were detected successfully. An overlap between a detected ROIs and a manually detected cell were designated True Positives (TP). ROIs that overlapped with multiple cells were not included in the TPs and were counted separately. Likewise, if ROIs did not match a manually detected cell or if multiple ROIs were assigned to one cell, the redundant ROIs were designated False Positives (FP). Cells not detected manually were included in the False Negatives (FN).

Using this classification, the correctness of the detection algorithm was assessed using three different measures: the true positive rate (TPR) or sensitivity by Eq. (13), the positive predictive value (PPV) using Eq. (14), and the recall defined as per Eq. (15).

(13) $$\text{TPR}=\text{Sensitivity}=\frac{\text{TP}}{P}$$

(14) $$\text{PPV}=\frac{\text{TP}}{\text{TP}+\text{FP}}$$

(15) $$\text{recall}=\frac{\text{TP}}{\text{TP}+\text{FN}}$$

### 3.3 Benchmark ROI detection

The performance of the cell detection on the same datasets was benchmarked against the alternative segmentation strategies. The active contours algorithm (FluoroSNNAP [13]) was studied for its ability to capture the crude shapes. The initial estimate made with the use of this algorithm did not require any user input.

Otsu thresholding (CellPRofiler [11]) was validated because of its ability to separate foreground and background pixels. A global threshold was chosen accepting ROIs diameter from 3 to 150 pixel units (pu) (Dataset 1), 25–250 pu (Dataset 2), and 10–45 pu (Dataset 3). A three-classes threshold was chosen, middle intensity pixels were assigned to the background and the threshold correction values were set to 1.3, 0.6, and 1.0 respectively for three datasets in question. In Dataset 1, a 50-pixel window adaptive threshold was chosen, the other datasets were processed with a global threshold. Merged cells were detected and separated based on pixel intensity.

We tested a combination of a thresholding and water shedding (ImageJ version 1.51k [16]) because of its speed. The image was converted to 8 bit to enable water shedding later on, the threshold was set to 15, 30, and 25 respectively (Datasets 1, 2, and 3). After water shedding was applied to separate the ROIs, only ROIs in the range of 50–500 pixels (Datasets 1), more than 80 pixels (2), and more than 10 pixels (3) were considered by using the Analyze Particles option.

Lastly, we performed the spatiotemporal independent component analysis (SIMA [10], [27] because this algorithm combines both spatial and temporal information to determine the cell's locations. The number of principal components used was chosen to be 500 for Dataset 1, 160 for Dataset 2, and 100 for Dataset 3, which is slightly higher than the estimated number of active cells [6]. The relative weight between the spatial and temporal independent component analysis was set to 0.85 (dominant spatial information) regarding the not-so-active Dataset 1 and the sparsely populated Dataset 3. In Dataset 2, this weight was set to 0.15. Cells overlapping more than 50% were merged and the ROIs were smoothed and filtered using the standard implementation of a method SparseROIsFromMasks proposed in SIMA [10].

All data were processed using an HP EliteBook 850 G2 with 16 GB physical memory running a 64-bit Windows 10 operating system to compare the results obtained using each method. The time required for estimating the segmentation was recorded for each segmentation strategy. Setting the parameters required about the same amount of time for each method; thus, it was ignored.

### 3.4 Benchmark spike detection and network analysis

The spikes detected by CALIMA were validated against the ones obtained through manual detection and compared to a waveform-based detection algorithm (FluoroSNNAP [13]). Dataset 3 was used to validate both the spike detection and the network reconstruction, as the optimal settings for spike detection were known and it had already been validated to contain neurons communicating in a network [13]. To avoid considering changes in the segmentations, the segmentation mask defined manually was used in both software tools before validating the spikes and network. The first 15 ROIs (indexes assigned by FluoroSNNAP) were used to check the spike detection.

The spikes were manually checked regarding height and shape, and sets of the cells matching the criteria were stored. In CALIMA, the spike detection was based on the mean values per ROI and the parameters were set to 10, 5, 0.50, and 0.100 for *l, z_th_, m*, and roc_min_ respectively. The relatively high value of *z_th_* is a direct result of the low amount of noise in Dataset 3. In FluoroSNNAP, the settings recommended by the authors were used: choosing a 10 s window and using the lower 50% of samples to determine the ∆ F/ F_0_, using their standard library to match and detect the Calcium events.

To reconstruct a network, CALIMA used the Pearson coefficients in combination with the inter-cellular distance. ROIs with a cross-correlation coefficient above 0.25, a distance lower than 208 µm (a full image width estimated at 1040 µm), and a response time of 500 ms or less to each other were considered in this case. FluoroSNNAP used a partial-correlation method with an alpha level of 0.001, as this method represents the inter-cell connections, which are the closest ones among all methods offered by the software [13].

## 4 Results

Using the methods as described in the previous section, the datasets described in Section 3.1 were processed using CALIMA and other alternative software tools. The results are shown in Table 1 and described in the following sections.

**Table 1:** Comparison of the assets and performance of different software tools on the given datasets.

Tool name	CALIMA	FluoroSNNAP	CellProfiler	ImageJ	SIMA
Segmentation strategy	Difference of Gaussians	Active contours	Otsu-thresholding	Thresholding & watershedding	Spatiotemporal ICA
Spike detection	Yes	Yes	Yes	No	No
Network analysis	Yes	Yes	No	No	No
Sensitivity range [%]	80–86	21–65	51–85	39–77	5–28
Positive prediction value range [%]	60–80	15–93	27–84	4–87	3.8–49
Recall [%]	88–98	39–69	79–99	41–94	41–100
Merged cells (Dataset 1,2,3)	45,14,14	13,71,4	0,72,6	6,87,4	159,143,10
Mean raw calculation time for segmentation [s]	5	11	5	40	1139

### 4.1 Cell detection

**Fig. 3:**
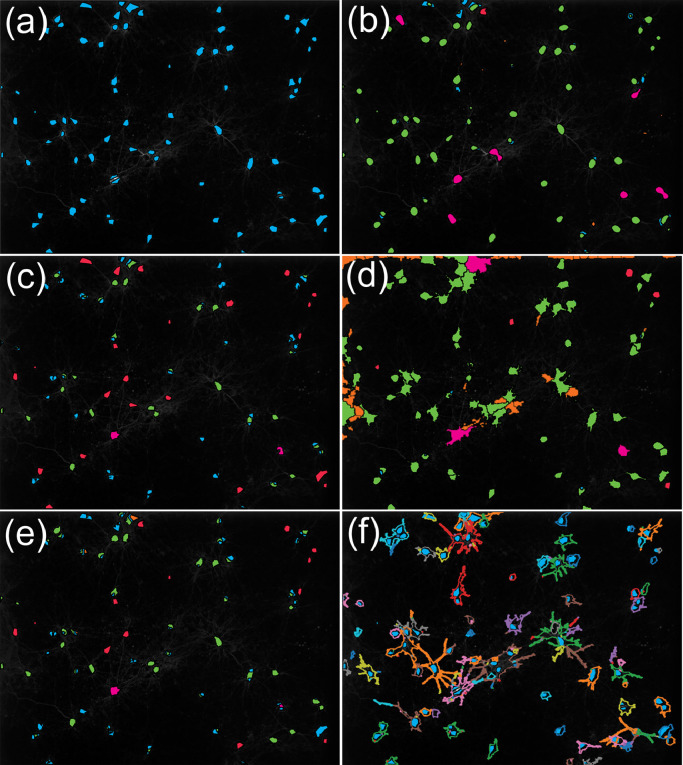
Detected ROIs for Dataset 3 using various segmentation strategies. ROIs, manual (a), ROIs, CALIMA (b), ROIs, Active Contours (FSNNAP) (c), ROIs, Otsu Thresholding + brightness-based declumping (CellProfiler) (d), ROIs, Thresholding + Watershedding (ImageJ) (e), ROIs, spatiotemporal Independent component analysis (SIMA) (f). The ROIs found manually are depicted in blue, the true positives in green, and the false positives in orange. The ROIs containing multiple cells are shown in purple and the false negatives in red. Note that an exception is made for the stICA method, as it allows regions to overlap. To avoid confusion, the segmentation outlines found by this method are shown as well.

**Fig. A.2:**
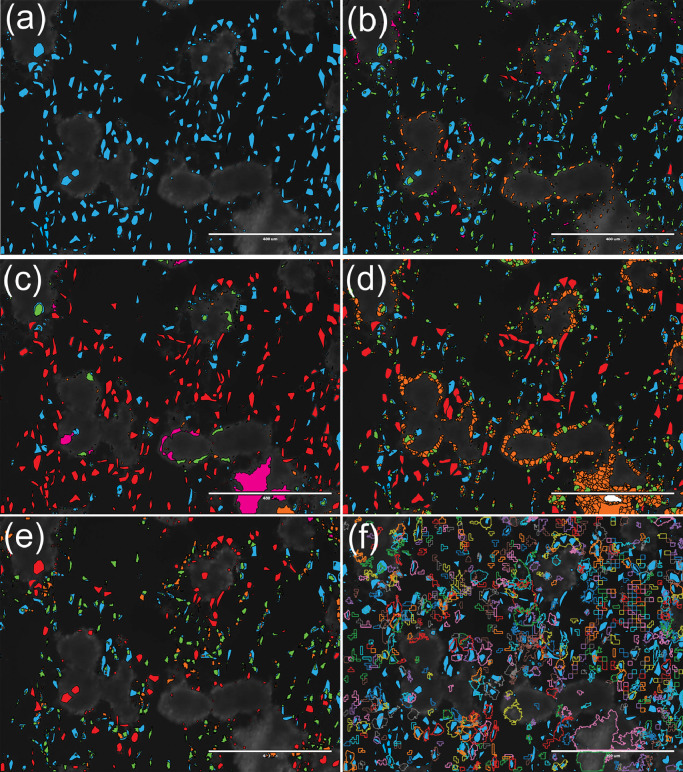
Detected ROIs for Dataset 1 using various segmentation strategies. The bar represents a width of 400 µm. ROIs, manual (a), ROIs, CALIMA (b), ROIs, Active Contours (FSNNAP) (c), ROIs, Otsu Thresholding + brightness-based declumping (CellProfiler) (d), ROIs, Thresholding + Watershedding (ImageJ) (e), ROIs, spatiotemporal Independent component analysis (SIMA) (f). The ROIs found manually are depicted in blue, the true positives in green, and the false positives in orange. The ROIs containing multiple cells are shown in purple and the false negatives in red. Note that an exception is made for the stICA method, as it allows regions to overlap. To avoid confusion, the segmentation outlines found by this method are shown as well.

The results of the cell detection accuracy across the three datasets are given in Table 1 (a more detailed analysis per dataset can be found in Table C.1.), and the ROIs found in dataset 3 are represented in Fig. 3 (ROIs found for datasets 1 and 2 can be found in Figs. A.2 and A.3). CALIMA demonstrates high scores for all three datasets with a sensitivity of 80–86% and a PPV of 60–80%. Though CALIMA performs well on the densely packed Dataset 2, the detection method comes at a cost. CALIMA detects only a part of the ROIs rather than the entire shape. Nevertheless, the raw calculation time is small, which helps a user to test different settings and find the optimum quickly.

The active contours algorithm appears to perform better in detecting the full extent of the ROIs but can detect only a smaller number of them. The Otsu-thresholding performs well in terms of sensitivity, speed, and determining the ROIs' actual shape to the cost of obtaining some redundant ROIs at the borders. Furthermore, when the cells are packed too densely (Dataset 2), the automated declumping has problems. The Thresholding and Watershedding strategy has difficulties with densely packed cells as well; however, it is quite sensitive in other cases. The separation of the cells appears to work only for small clusters of cells, as indicated by a large number of merged cells in Dataset 2. The stICA method appears to scatter its ROIs, resulting in obtaining many isolated small and overlapping regions. However, stICA shows the best performance among all considered algorithms in terms of finding the ROI outlines, and it is able to detect shapes as small as neurites. An option to automatically detect cells of different shapes will be considered as well for CALIMA in the future [28].

### 4.2 Spike detection and network analysis

Both the z-score algorithm and the database comparison demonstrated appropriate scores in terms of sensitivity; FluoroSNNAP was able to identify 96.5% of the peaks on average (ranging between 90 – 100% per cell) and CALIMA identified 98.5% (84–100%). The database comparison for FluoroSNNAP had 32 false positives spread over 9 cells, while CALIMA discovered 14 in 6 cells. Most false positives of the database comparison (16) were found in a single cell indicating the inhibition of a brightness drift and a large number of non-AP spikes. The Z-score's false positives were spread more evenly across the cells. The number of false negatives of FluoroSNNAP was 18 across 9 cells and for CALIMA 19 across 9 cells. The Z-score algorithm missed most of peaks (6) in a single cell; this observation indicates fast spiking activity.

**Fig. 4:**
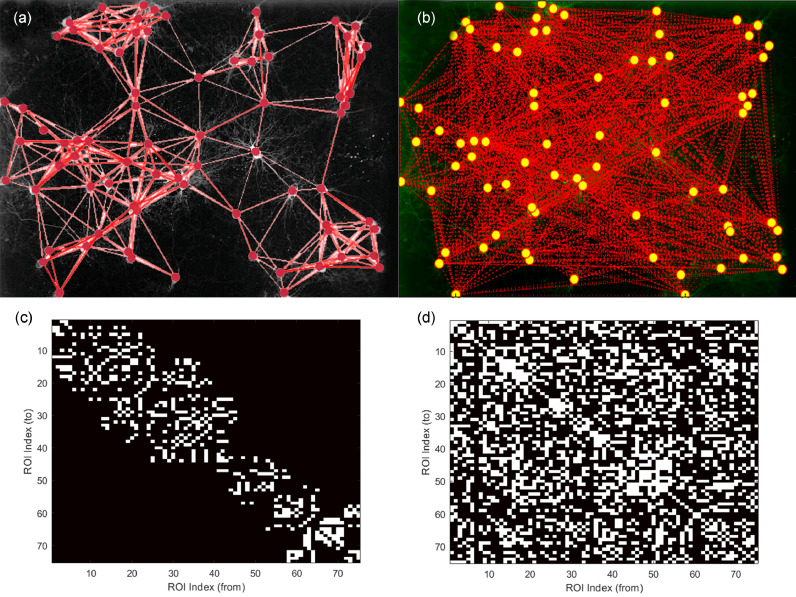
The reconstructed networks and binary adjacency matrices for dataset 3. Reconstructed network, ROI Pearson cross-correlation + distance filter (CALIMA) (a). Reconstructed network, ROI partial correlation (FSNNAP) (b).The ROIs are shown as dots and the connections as lines. Binary adjacency matrix of CALIMA (c) and FSNNAP (d). The matrices represent the ROIs, those that are connected (in white) and those that are not.

As can be seen from Fig. 2a and b, the networks reconstructed using the 2 different methods differ significantly from each other (Fig. 4c and d). In addition, the binary adjacency matrices also differ, with much less ROIs being linked with one another. The most typical difference is that the connections in CALIMA are shorter, while FluoroSNNAP allows observing connections over larger distances. Since a typical neurite length is in the order of 100′s µm [29], the user-defined maximum length of neuronal connections allows CALIMA to filter out these longer connections, resulting in a physiologically more sensible communication network including both functional and anatomical parameters.

## 5 Discussion

CALIMA was tested on three real-life datasets; consequently, it demonstrated a high sensitivity for the ROIs and allowed performing quick and highly confident cell detection on a large variety of data. When compared with other segmentation strategies, CALIMA performed better in most cases (see Tables 1 and C1).

The automatic mode of the active contours algorithm (FluoroSNNAP) allows detecting only a small number of ROIs. This algorithm performs better if the user provides growth regions. Otsu's method (CellProfiler) enables multiple-class thresholds that can be combined with an adaptive window. Though the algorithm detects most cells correctly as well as identifies the ROI shape in detail, it does not perform well when cells are densely packed. As a result, the algorithm might merge a number of cells. The ‘declumping’ algorithm would have to be fine tuned by the user to correct this. The thresholding strategy (ImageJ) works well to detect large clusters of cells, but it is inaccurate when it makes segmentation of these clusters into separate cells as a next step. The cell irregular shapes are the likely cause of this. The algorithm has a problem with detecting cells near the cellular debris, which is because these cells are merged in a layer of moderate brightness, which cannot easily be handled by using a single threshold. Lastly, the stICA method (used in SIMA) does not show good results in terms of the sensitivity of its performance, or the redundant areas it discovers. Dataset 1 does not show sufficient activity for the algorithm to find the separate cells, and in Dataset 2 the cells are packed too densely. Therefore, it was concluded that stICA, while being able to detect the cellular shape up to the detail level of the neurites, is not suitable to analyze such cultures as ones presented in Datasets 1 and 2 and requires a low culture cell density to successfully analyze the footage, which is not the case for CALIMA.

Using the correct parameters, CALIMA's Z-score algorithm is able to detect a large number of calcium-spikes correctly. A sample set of 15 cells demonstrated a sensitivity range of 86–100%. These values are comparable to the results observed for the active contours algorithm (FluoroSNNAP). However, it was concluded that the FluoroSNNAP algorithm exhibits difficulties when separating between action potentials and spike-mimicking noise. Meanwhile, the z-score algorithm of CALIMA has problems with identifying all spikes in cells showing near-constant activity. Although these are rare events, the excess of spikes disrupts the estimation of the standard deviation that is required by CALIMA to detect these spikes correctly.

The networks reconstructed by both CALIMA and FluoroSNNAP differ at various points; the lengths of the connections as well as the actual connections differ. This is probably a result of the different reconstruction strategies implemented. CALIMA focuses on the physical limitations of the cells when identifying the network structure and tries to establish the actual connections. FluoroSNNAP rather focuses on the transfer of information within the network and links all blinking cells together. As a result, CALIMA reconstructed network is a physiologically more sensible communication network including both functional (similar synchronous blinking pattern) and anatomical parameters (physical distance between neuron).

## 6 Conclusion

Although calcium imaging is widely used in biological research, there are relatively few free and automated tools available for performing such analysis. These tools often enable only part of the analysis. Here we proposed the standalone free and open-source software tool CALIMA, and we demonstrated that it could serve as a valuable addition to the field of calcium imaging. Starting from crude calcium imaging data, CALIMA can detect cells, determine their activity, and reconstruct a communication network within minutes.

As it was proved that CALIMA is able to detect ROIs, find Ca-peaks, and establish a communication network, we consider CALIMA to be a valuable addition to the existing set of Ca-analysis tools. Because the DOG algorithm detects only a small part of most ROIs, future versions of CALIMA will contain a region-growing step to complete identification process for those areas for which only a part has been found. An option to automatically detect cells of different shapes will be considered as well. Furthermore, the algorithm detecting the physical network will be combined with information transfer theories to study the communication between cells in detail.

## Conflict of interest

The authors declare that the research was conducted in the absence of any commercial or financial relationships that could be construed as a potential conflict of interest. This statement has also been added at the end of the manuscript.

## Figures and Tables

**Fig. A.3:**
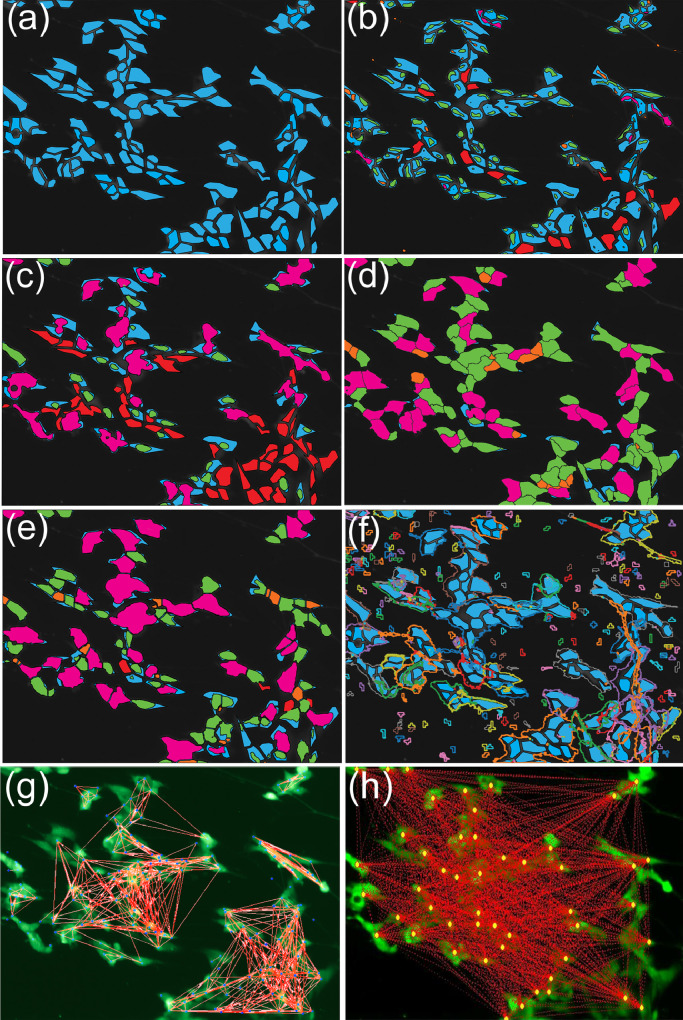
Detected ROIs for Dataset 2 using various segmentation strategies. ROIs, manual (a), ROIs, CALIMA (b), ROIs, Active Contours (FSNNAP) (c), ROIs, Otsu Thresholding + brightness-based declumping (CellProfiler) (d), ROIs, Thresholding + Watershedding (ImageJ) (e), ROIs, spatiotemporal Independent component analysis (SIMA) (f). The ROIs found manually are depicted in blue, the true positives in green, and the false positives in orange. The ROIs containing multiple cells are shown in purple and the false negatives in red. Note that an exception is made for the stICA method, as it allows regions to overlap. To avoid confusion, the segmentation outlines found by this method are shown as well. The reconstructed networks and binary adjacency matrices for dataset 2 are also shown. Reconstructed network, ROI Pearson cross-correlation + distance filter (CALIMA) (g). Reconstructed network, ROI partial correlation (FSNNAP) (h).

**Table B.1:** The variables used for cell detection for the various datasets.

σn	σa	σb	th	
Dataset 1	4	1	4	0.001
Dataset 2	6.2	1	3	0.0001
Dataset 3	6.5	1	2.7	0.0002

**Table C.1:** The total number of cells found in the dataset, the number of true positives, false negatives, merged ROIs, and the number false positive ROIs per segmentation strategy. The detection sensitivity, positive predictive value and recall are shown in the last three columns. The best score per dataset is marked bold.

Dataset1	Total	TP	FN	Merged	FP	Detec Rate	PPV	Recall
Difference of Gaussians (CALIMA)	488	417	26	45	277	0.85	0.6	0.94
Active contours (Fluorosnnap)	488	184	291	13	46	0.38	0.8	0.39
Otsu-thresholding + declumping (CellProfiler)	488	387	101	0	1053	0.79	0.27	0.79
Thresholding + watershedding (ImageJ)	488	285	197	6	187	0.6	0.39	0.59
SpatioTemporal ICA (SIMA)	488	134	195	159	290	0.27	0.32	0.41

Dataset2	Total	TP	FN	Merged	FP	Detec Rate	PPV	Recall
Difference of Gaussians (CALIMA)	150	120	16	14	16	0.8	0.88	0.88
Active contours (Fluorosnnap)	150	31	48	71	175	0.21	0.15	0.39
Otsu-thresholding + declumping (CellProfiler)	150	72	1	77	15	0.5	0.83	0.99
Thresholding + watershedding (ImageJ)	150	59	4	87	16	0.39	0.79	0.94
SpatioTemporal ICA (SIMA)	150	7	0	143	175	0.05	0.04	1

Dataset3	Total	TP	FN	Merged	FP	Detec Rate	PPV	Recall
Difference of Gaussians (CALIMA)	75	60	1	14	12	0.8	0.83	0.98
Active contours (Fluorosnnap)	75	49	22	4	4	0.65	0.92	0.69
Otsu-thresholding + declumping (CellProfiler)	75	64	5	6	43	0.85	0.6	0.93
Thresholding + watershedding (ImageJ)	75	58	13	4	9	0.77	0.87	0.82
SpatioTemporal ICA (SIMA)	75	49	1	25	51	0.65	0.49	0.98

## Appendix D Supplementary materials

Image, application 1
